# Supplementary figures and images for: Factors associated with resistance to SARS-CoV-2 infection discovered using large-scale medical record data and machine learning
Source: PLoS One. 2023 Feb 22;18(2):e0278466. doi: 10.1371/journal.pone.0278466 (PMC9946212; doi:10.1371/journal.pone.0278466)

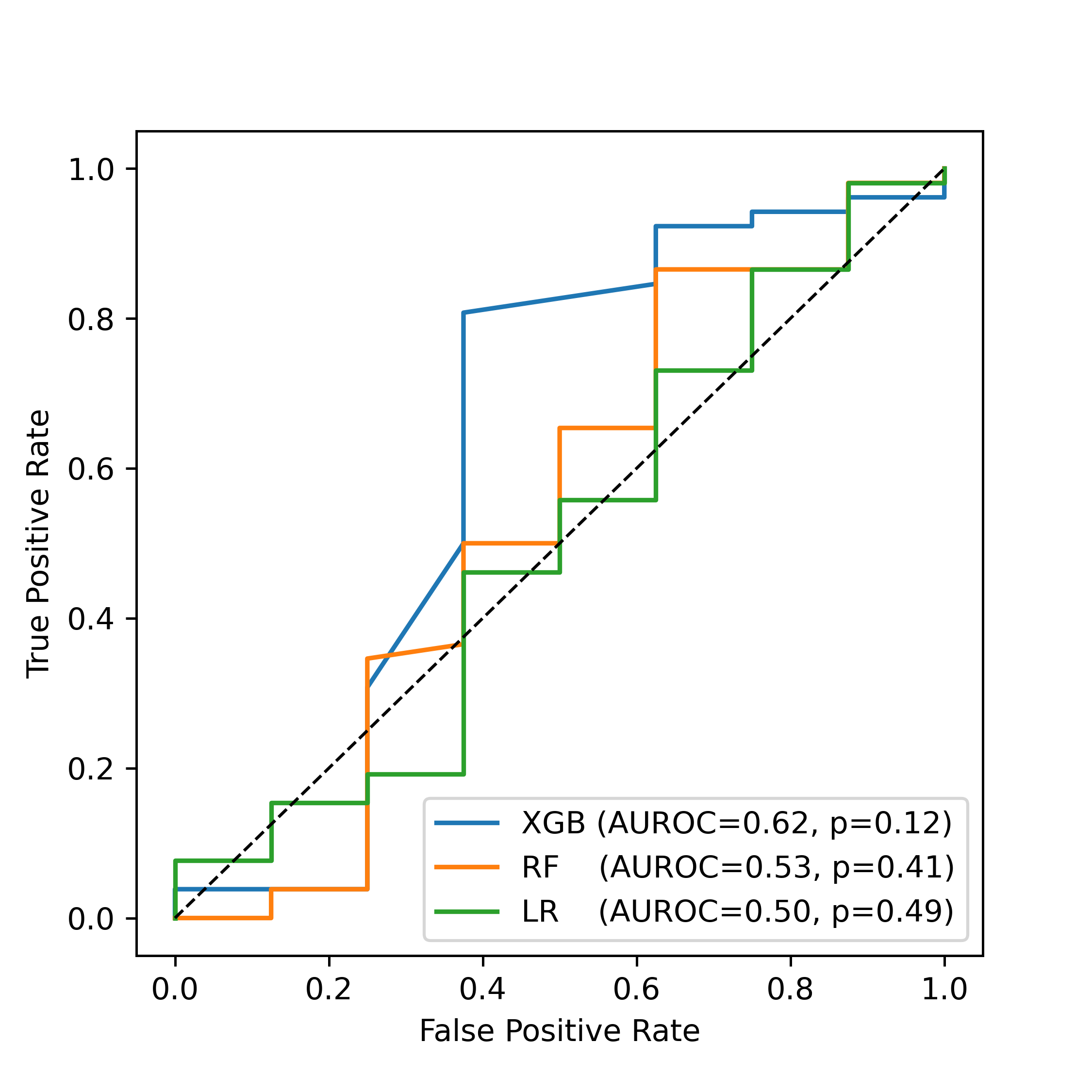

Supplement: S1 Text — (ZIP) [file pone.0278466.s001.zip › Fig/AUROC_HH_testing.png]

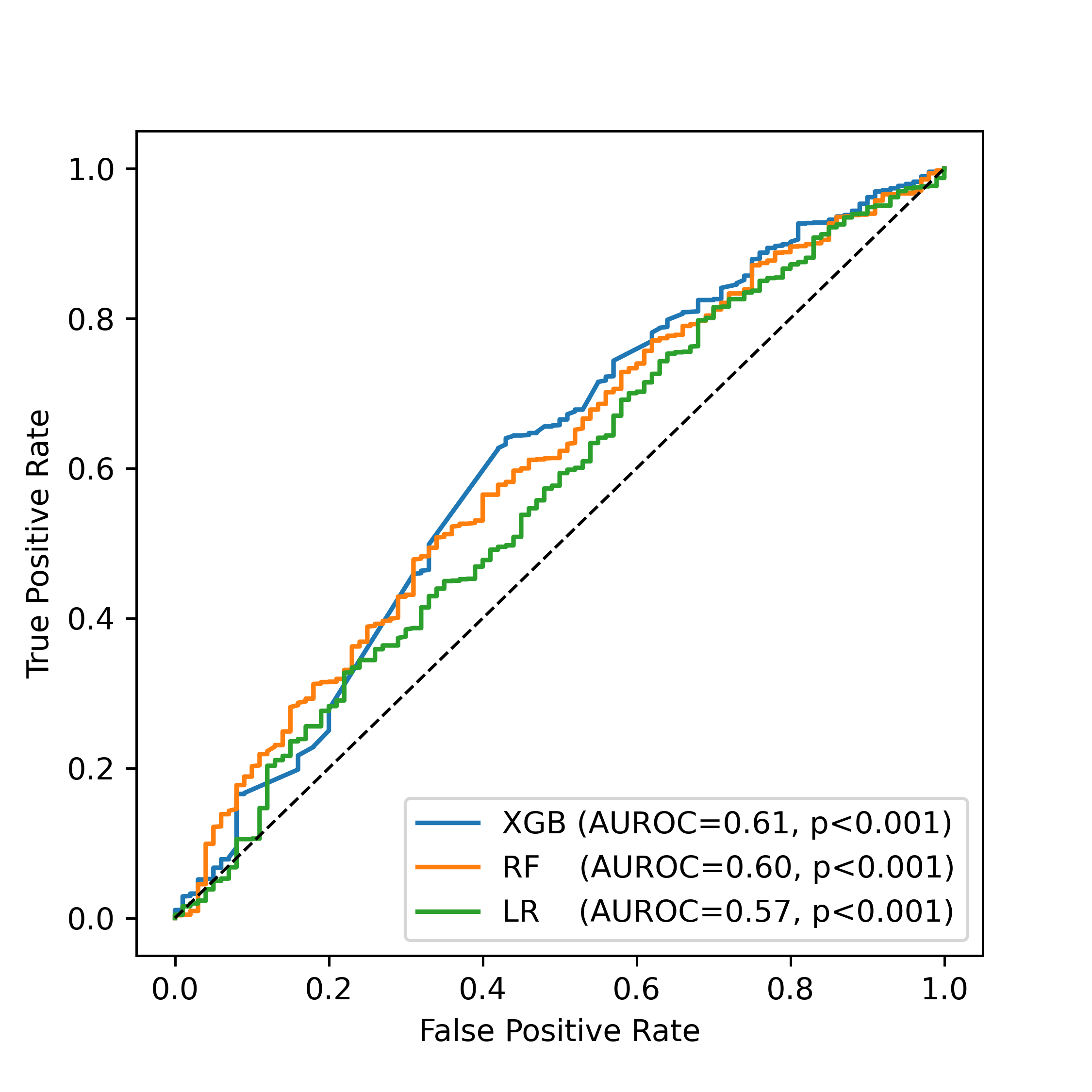

Supplement: S1 Text — (ZIP) [file pone.0278466.s001.zip › Fig/AUROC_testing.png]

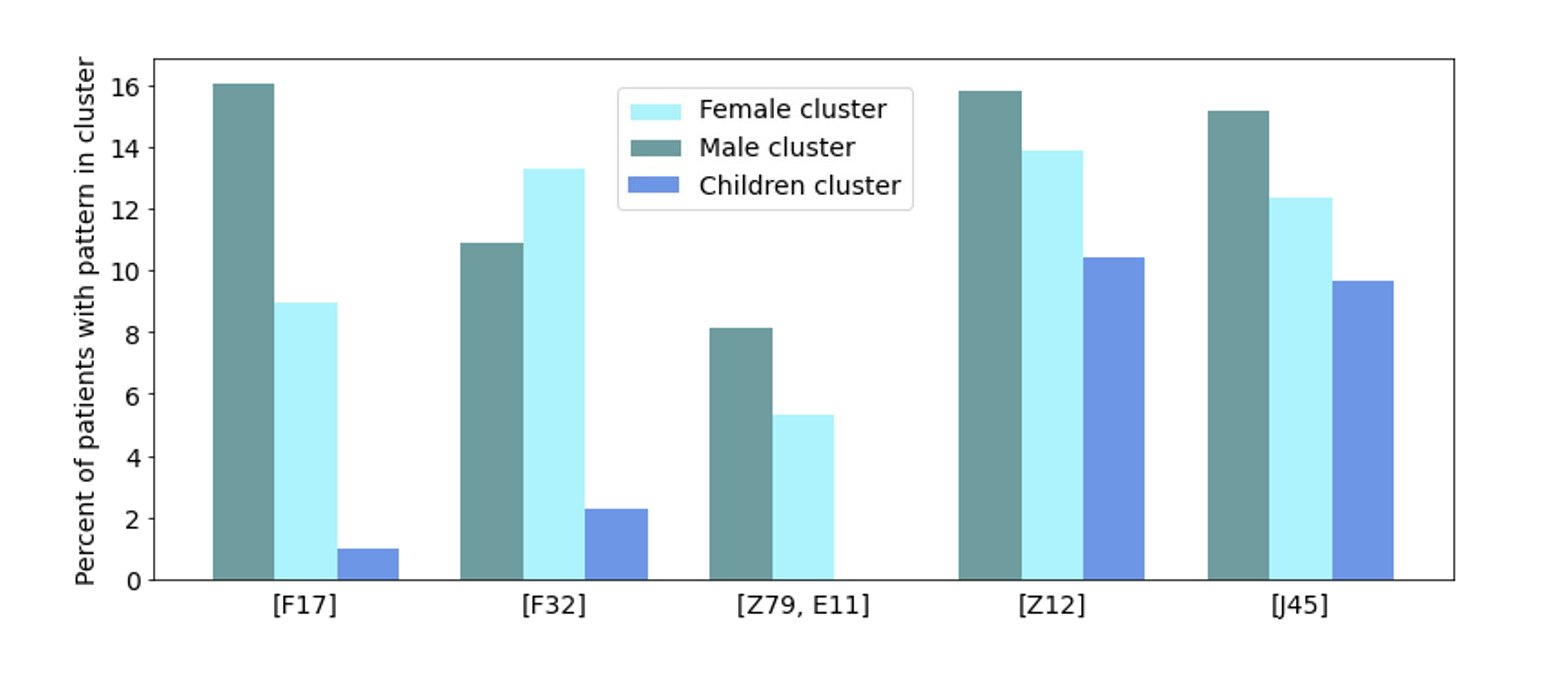

Supplement: S1 Text — (ZIP) [file pone.0278466.s001.zip › Fig/clustering_results.png]

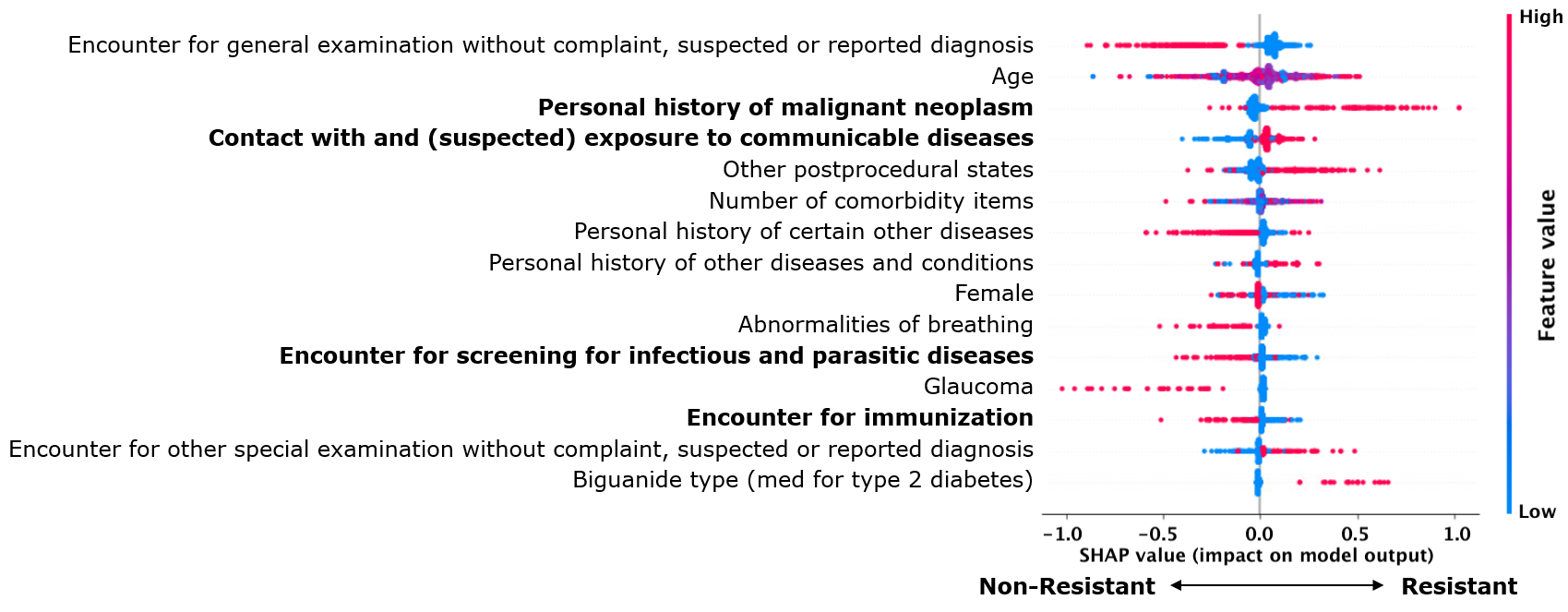

Supplement: S1 Text — (ZIP) [file pone.0278466.s001.zip › Fig/Shap.png]

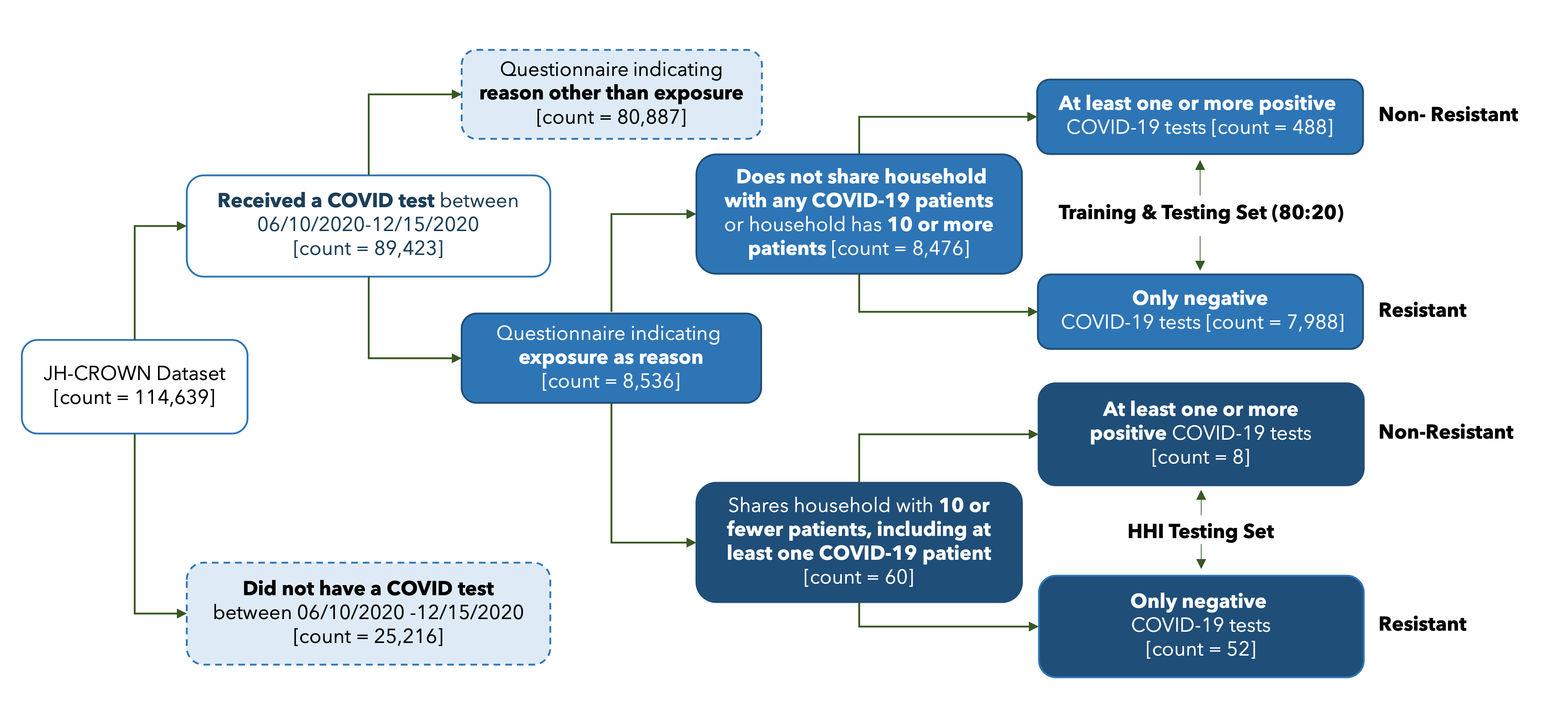

Supplement: S1 Text — (ZIP) [file pone.0278466.s001.zip › Fig/Patient Cohort Selection.png]

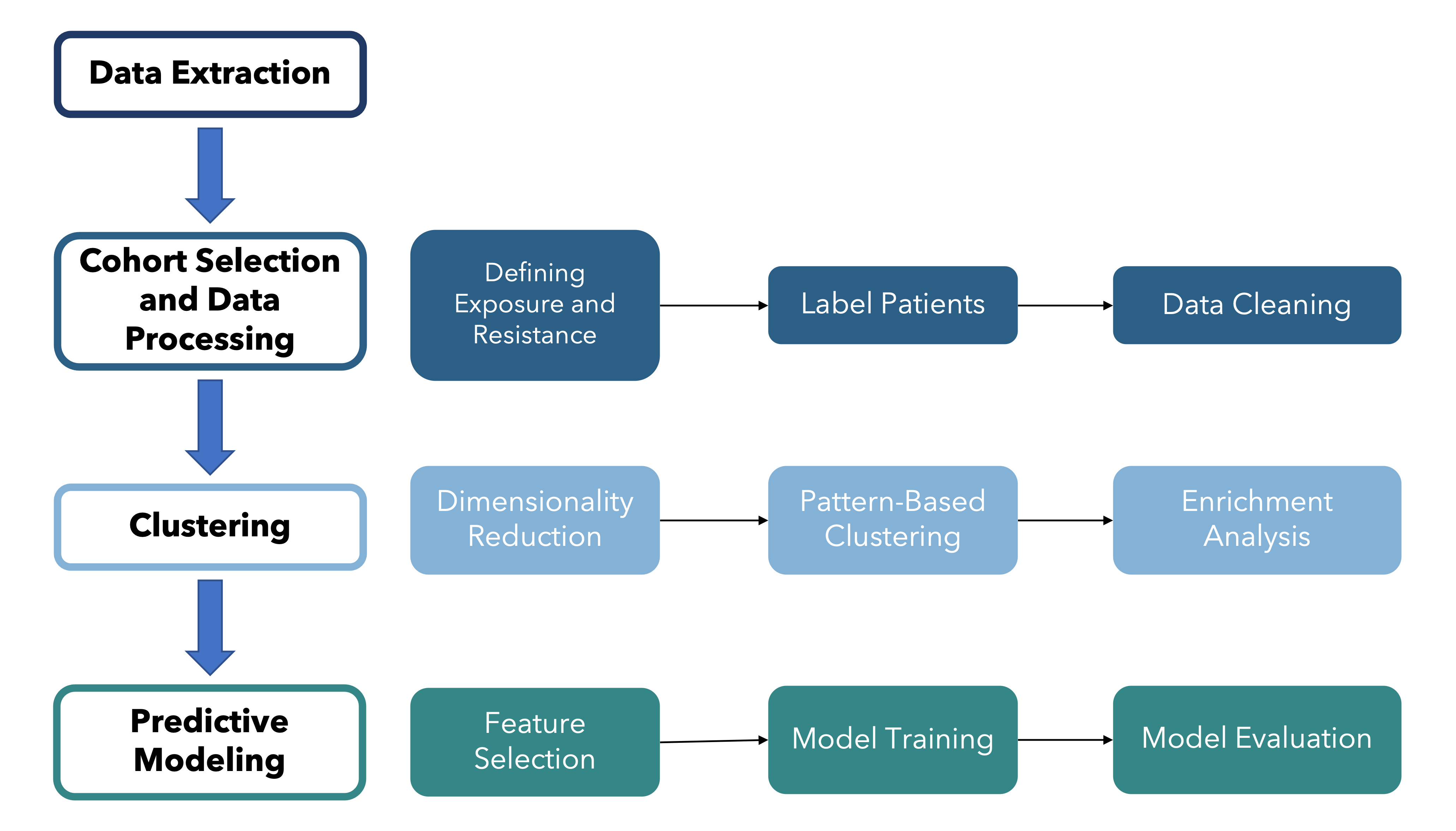

Supplement: S1 Text — (ZIP) [file pone.0278466.s001.zip › Fig/Workflow Graph.png]
