# Supplementary material for: Factors associated with resistance to SARS-CoV-2 infection discovered using large-scale medical record data and machine learning
Source: PLoS One. 2023 Feb 22;18(2):e0278466. doi: 10.1371/journal.pone.0278466 (PMC9946212; doi:10.1371/journal.pone.0278466)
Supplement: Attachment — Submitted filename: PLOS ONE_Respond to Reviewers.pdf [file pone.0278466.s002.pdf]

Original Manuscript ID: PONE-D-22-23813

Original Article Title: Factors Associated with Resistance to SARS-CoV-2 Infection Discovered Using Large-scale Medical Record Data and Machine Learning

To: PLOS ONE Editor

Re: Response to reviewers

Dear Editor,

Thank you for allowing us to resubmit our manuscript and giving us an opportunity to address the reviewers' comments. We genuinely appreciate the thorough review of the manuscript by the reviewers. Your constructive feedback and suggestions have been very helpful in the revision and betterment of this paper.

For this resubmission, we are uploading the following:

(1) Our point-by-point response to the comments (in **blue fonts** below) (response to reviewers and editors).

(2) An updated manuscript with the **blue fonts** highlighting indicated changes (PCM_PLOS_Manuscript_track)

(3) A clean updated manuscript without highlights (PCM_PLOS_Manuscript_clean)

Best regards,

Kai-Wen K. Yang, Rebecca Yoo, Ilya Rattsev, Chloé Paris, Kevin Gorman, Yijia Chen, Jacob M. Desman, Tony Y. Wei, Joseph L. Greenstein, Casey Overby Taylor, Stuart C. Ray

I. Comments from Editor

1. **Concern #1:** Please ensure that your manuscript meets PLOS ONE's style requirements, including those for file naming. The PLOS ONE style templates can be found at https://journals.plos.org/plosone/s/file?id=wjVg/PLOSOne_formatting_sample_main_body.pdf and https://journals.plos.org/plosone/s/file?id=ba62/PLOSOne_formatting_sample_title_authors_affiliations.pdf

   Thank you for the reminder. We have cross-checked our manuscript with the PLOS ONE style template for both the main body and title authors affiliations. We confirmed that it aligns with the style requirements stipulated therein.

2. **Concern #2:** We note that the grant information you provided in the 'Funding Information' and 'Financial Disclosure' sections do not match. When you resubmit, please ensure that you provide the correct grant numbers for the awards you received for your study in the 'Funding Information' section.

   Thank you for noticing this! We have made sure that the information regarding the funding information and financial disclosure sections are aligned.

3. **Concern #3:** Thank you for stating the following financial disclosure:
   "The data utilized were part of JH-CROWN: The COVID PMAP Registry, which is based on the contribution of many patients and clinicians and is funded by Hopkins inHealth, the Johns Hopkins Precision Medicine Program. Project-specific costs of data extraction were defrayed by funds from the Office of the Dean, JHU School of Medicine." Please state what role the funders took in the study. If the funders had no role, please state: "The funders had no role in study design, data collection and analysis, decision to publish, or preparation of the manuscript." If this statement is not correct you must amend it as needed. Please include this amended Role of Funder statement in your cover letter; we will change the online submission form on your behalf.

   We confirm that the funders had no role in our study and have included the below amended role of funder statement in our cover letter:

   > "The funders had no role in study design, data collection and analysis, decision to publish, or preparation of the manuscript."

4. **Concern #4:** Thank you for stating the following in the Acknowledgments Section of your manuscript:
   "The data utilized were part of JH-CROWN: The COVID PMAP Registry, which is based on the contribution of many patients and clinicians and is funded by Hopkins inHealth, the Johns Hopkins Precision Medicine Program. Project-specific costs of data extraction were defrayed by funds from the Office of the Dean, JHU School of Medicine."

   We note that you have provided funding information that is not currently declared in your Funding Statement. However, funding information should not appear in the Acknowledgments section or other areas of your manuscript. We will only publish funding information present in the Funding Statement section of the online submission form.

   Please remove any funding-related text from the manuscript and let us know how you would like to update your Funding Statement. Currently, your Funding Statement reads as follows:
   "The data utilized were part of JH-CROWN: The COVID PMAP Registry, which is based on the contribution of many patients and clinicians and is funded by Hopkins in Health, the Johns Hopkins Precision Medicine Program. Project-specific costs of data extraction were defrayed by funds from the Office of the Dean, JHU School of Medicine."

   Please include your amended statements within your cover letter; we will change the online submission form on your behalf.

   Thank you for the clarification. We have:

   (1) removed any funding-related information from the Acknowledgements section and any other areas of our manuscript. The updated acknowledgement section is as follows:

   > "We wish to thank Dr. Dhananjay Vaidya, Dr. Jacky Jennings, Lisa Yanek, and Bahareh Modanloo from the Biostatistics, Epidemiology, and Data Management (BEAD) core group for their guidance and constructive comments on study design and analysis approaches. We also thank Kerry Smith and Michael Cook from the Center for Clinical Research Data Acquisition (CCDA) for their help in data

extraction and data navigation. The data utilized were part of JH-CROWN: The COVID PMAP Registry, which is based on the contribution of many patients and clinicians.

(2) included our amended Funding Statement in the cover letter, which is as follows:
"JH-CROWN received funding from Hopkins inHealth, the Johns Hopkins Precision Medicine Program. Project-specific costs of data extraction were defrayed by funds from the Office of the Dean, JHU School of Medicine."

5. **Concern #5:** In your Data Availability statement, you have not specified where the minimal data set underlying the results described in your manuscript can be found. PLOS defines a study's minimal data set as the underlying data used to reach the conclusions drawn in the manuscript and any additional data required to replicate the reported study findings in their entirety. All PLOS journals require that the minimal data set be made fully available. For more information about our data policy, please see http://journals.plos.org/plosone/s/data-availability.

"Upon re-submitting your revised manuscript, please upload your study's minimal underlying data set as either Supporting Information files or to a stable, public repository and include the relevant URLs, DOIs, or accession numbers within your revised cover letter. For a list of acceptable repositories, please see http://journals.plos.org/plosone/s/data-availability#loc-recommended-repositories. Any potentially identifying patient information must be fully anonymized.

Important: **If there are ethical or legal restrictions to sharing your data publicly, please explain these restrictions in detail.** Please see our guidelines for more information on what we consider unacceptable restrictions to publicly sharing data: http://journals.plos.org/plosone/s/data-availability#loc-unacceptable-data-access-restrictions. Note that it is not acceptable for the authors to be the sole named individuals responsible for ensuring data access.

We will update your Data Availability statement to reflect the information you provide in your cover letter.

Thank you for pointing this out and providing detailed guidelines for us! We have read through the data policies and restrictions in the links you provided, and we have concluded that it will not be possible for us to release a minimal dataset as we are using third-party data. Furthermore, if we deidentified our data according to minimal dataset guidelines, our analysis would be impossible to replicate as time and location are involved in the analysis, so using date shifting and/or generalizing time points to years would not be feasible when reproducing our results. Instead, we will follow the Data Availability Statement guidelines required for third-party data.

Below is the Data Availability statement that can be included in the submission:
"Our dataset cannot be shared publicly due to IRB restrictions on data obtained from participants without consent for sharing publicly. Researchers who wish to collaborate in analysis of JH-Crown data will need to collaborate with a Johns Hopkins University investigator and obtain IRB approval. Those who are requesting access to the JH-CROWN data should contact the PI, Dr. Garibaldi, at bgariba1@jhmi.edu."

6. **Concern #6:** Please include a caption for figure 3a and 3b.

We apologize for missing that. We have revised our manuscript and included captions for 3a and 3b (4a and 4b for the updated manuscript). We have also confirmed that all figures in the resubmitted manuscript have captions.

II. Comments from Reviewer #1:

1. **Concern #1:** This project begins with a worthy question - why do some people get exposed to COVID, but not develop SARS-COV-2. The writing is good and the figures are fine. Unfortunately, the data and analysis do not meet the needs of question. As the limitations section (appropriately) makes clear, there is simply an untenable amount of noise and bias in every variable in the dataset to answer this question. The exposure, outcome, and key predictors are all absolutely unreliable to address the underlying question they are hoping of them, given low testing rates, poorly-measured exposure, etc. ICD codes are rarely accurate, but especially given the crazy and unreliable primary care access that we all experienced in 2020. I just don't trust these data for these questions.

Thank you for raising these important points. As you mentioned, we do recognize that EHR data have these various limitations and made it clear in our limitations section (as shown below), citing the self-reported

nature of exposure, the poor-capture of degree and duration of exposure, the difficulty in creating a high exposure cohort, and the inherent problems associated with a limited timeframe among other limitations.

> "Exposure to SARS-CoV-2 was self-reported through the COVID-19 testing questionnaire by the participant, and therefore, is a subject to reporting bias and is not completely reliable. The degree and duration of the exposure are not well-captured, and it is impossible to tell whether a subject was wearing personal protective equipment (e.g., mask) during the exposure, which could have protected the individual from infection."

We still chose to use EHR data despite these limitations as we believe that it is possible to extract valuable information. There has been a wide range of research done on EHR data, and questions have been answered even with the noise and bias present. In addition, since EHR data is what we intend the algorithm to be used with, if the algorithm were to be translated to the clinic, we believe it is necessary for the algorithm to be developed on EHR data. We hope that this algorithm can be used by clinicians to make key decisions for preventive measures in future pandemics and we believe the discriminative ability of the model using these imperfect covariates can achieve this.

We also would like to note that we chose the most conservative methods so that our algorithm would be using data most similar to the data available in practice. In addition, we used ICD-10 codes which are billing codes and thus help with the reliability of our data. We have included this distinction in our revised manuscript with the following sentence:

> "We note that we chose to use ICD-10 codes, which are used for billing, as we believe they help making our data more reliable."

We do recognize that EHR data has noise and bias, and we hope that in the future, new methods will be developed to process the data and improve model performance. However, we believe that even with the current methods available, it is still possibility to answer our underlying question.

2. **Concern #2:** I was also concerned by the analysis, which had a lot of examples of what seemed to me to be opportunities for overfitting. These include running the same analysis with multiple different resampling and model-building techniques and multiple different tuning parameters, and using backward elimination without penalization. I am only too aware that this is common in machine learning, but it's a method of performing multiple comparisons without appropriate adjustment Similarly, "using five-fold validation to help tune the hyperparameters " has the same problem. Cross-validation is a validation technique, not a tuning technique.

Thank you for raising your concerns regarding the statistical approach we chose for the analysis. We fully acknowledge the limitations of machine learning approaches that you mentioned. However, given our study objective to predict COVID-19 resistance status in exposed individuals, we believe that machine learning was the best approach to tackle that question. We used a standard machine learning pipeline to find an optimal model and minimize the possibility of overfitting.

We realize that we did not mention the regularization approach we used in recursive feature elimination; we used L2 regularization technique during the recursive feature elimination step. The following sentence was added to the methods section to clarify this:

> "To reduce feature dimension, recursive feature elimination with L2 regularization was utilized to select the top 108 features (out of 1,310 features) to be included in model training."

Finally, all the steps that were a part of model optimization, including testing different resampling techniques and hyperparameter tuning, were performed exclusively on the training set. We then used optimized models to classify the subjects in the independent test set. Given that the final AUCs obtained while deploying the models on the independent testing set are comparable to the AUCs obtained during the model development on the training set, we suggest that the models were likely not overfitting.

3. **Concern #3:** Finally, I don't really understand the purpose the main goals of the analysis. For the classification model, what would we do with an effective classification tool? I'm not expecting this to be the end-all classification model, but it's nice to know why we're making a prediction.

Thank you for raising your concerns regarding the clinical utility of the model. We realize that we did not communicate it well in the main text, and added the following paragraph to the Discussion section to explain the potential use cases of an effective model:

"This work shows the feasibility of machine learning approaches to detect patients who are likely to be resistant to an emerging infection. A model with good discriminative ability may be used by public health professionals to enable better stratification of risk groups and improve surveillance. Similar tools have been developed previously for prediction of COVID-19 severity to improve care for patients hospitalized due to COVID-19. Given the high burden that was put onto the healthcare system early in COVID-19 pandemic, we believe that the resistance prediction tool may help allocate limited healthcare resources more efficiently in case of potential future outbreaks."

4. **Concern #4:** Similarly, I don't really understand what we hoped to learn from the clustering. Were we hoping to find a clear biologic cause of resistance? To me, a cluster implies actual meaningful differences between groups, like Type 1 vs. Type 2 diabetes. Clustering algorithms like this are designed to find clusters in what are usually actually just putting lines around non-meaningful differences. I'm not convinced these clusters are meaningful and I'm not sure what to do with them if they are. I do look forward to seeing further work in this field to understand why some patients did not develop SARS-COV-19.

   We used clustering for two main objectives: (1) exploratory analysis of the data, and (2) dimensionality reduction prior to performing association tests. As you noted in the comment, ideally, clustering analysis would produce some results that would help us explain the biological basis of resistance. However, we believed that the mechanism of resistance was too complex for clear features to be detected. It is likely that the interaction of features with each other explains resistance better than individual features do. As expected, the results of clustering did not reveal any clear clinical characteristics underlying resistance. However, the negative result showed us that more complex analysis needed to be done to find clinical factors associated with resistance.

   In addition, the patterns detected in the first phase of the MASPC algorithm were further used in association analysis to find the combinations of diagnostic codes and medications associated with resistance status. That way, we were able to remove sparse features from the enrichment analysis and adjust for correlation between diagnostic codes and medications. We added the following sentence in the Methods section ("Pattern selection and clustering" subsection) to clarify how the enrichment analysis was performed:

   "We then compared the prevalence of identified patterns in resistant and non-resistant individuals in an enrichment analysis. Fisher's exact test was used to estimate significance of the differences between cohorts ($\alpha = 0.05$)."

III. Comments from Reviewer #2:

1. **Concern #1:** The work seems to be very much appreciable. I have only a few points. 1. feature selection: why did not the authors mention the algorithm?

   Thank you for your comment regarding the algorithm we used for feature selection. We mentioned the algorithm used to select features for machine learning in the "Predictive model building" subsection of the Methods in the following sentence:

   "To reduce feature dimension, recursive feature elimination with L2 regularization was utilized to select the top 108 features (out of 1,310 features) to be included in model training."

   We realize that the subsection labeling is misleading, as we had a separate subsection called "Feature selection" that talks about initial preprocessing of the raw data. We changed the label of that subsection to "Data processing" to avoid this confusion.

2. **Concern #2:** A few more recent references to be added

   Thank you for the suggestion. We have added more recent references related to our topic.

   - This paper suggests that natural resistance against pathogens is found in many infections, and thus, resistance against SARS-CoV-2 may also exist. Understanding the mechanisms of natural resistance to COVID-19 can help us advance preventive measures against infections and identify potential therapeutic targets to treat COVID-19.
     Reference: Netea, M. G., Domínguez-Andrés, J., van de Veerdonk, F. L. et al. Natural resistance against infections: focus on COVID-19. Trends Immunol 43(2), 106–116 (2022). https://doi.org/10.1016/j.it.2021.12.001

- This study found that decreased OAS1 expression due to a common haplotype contributes to COVID-19 severity.
  Reference: Banday, A.R., Stanifer, M.L., Florez-Vargas, O. et al. Genetic regulation of OAS1 nonsense-mediated decay underlies association with COVID-19 hospitalization in patients of European and African ancestries. Nat Genet 54, 1103–1116 (2022). https://doi.org/10.1Re038/s41588-022-01113-z

- This study found that immune memory to common cold coronaviruses influences SARS-CoV-2 infection outcome.
  Reference: Yu, E. D., Narowski, T. M., Wang, E. et al. Immunological memory to common cold coronaviruses assessed longitudinally over a three-year period pre-COVID19 pandemic. Cell Host & Microbe 30(9), 1269-1278 (2022). https://doi.org/10.1016/j.chom.2022.07.012

- This study found that asthma does not increase the risk of SARS-CoV-2 infection while food allergy is associated with lower infection risk.
  Reference: Seibold. M. A., Moore, C. M., Everman, J. L. et al. Risk factors for SARS-CoV-2 infection and transmission in households with children with asthma and allergy: A prospective surveillance study. J. Allergy Clin. Immunol 150(2), 302-311 (2022). https://doi.org/10.1016/j.jaci.2022.05.014

3. **Concern #3:** questionnaire to be placed in Appendix.

   We have added the questionnaire and the possible answers to the Supporting information section (as shown below), thank you for the suggestion.

   > "We use responses to the EHR prompt, "What is the reason for testing this patient" in the JH-CROWN COVID-19 testing participant survey as part of our cohort selection. The possible responses are as follows, "Pre-procedure", "Hospital admission", "Surveillance screening program", "COVID exposure", "Other facility admission", "Delivery anticipated (will flag as PUI)", "To remove from COVID isolation (will flag as PUI)", "Pre-BMT/Organ transplant", "JHM Approved Travel", "Non-elective admission from a nursing home (will flag as PUI)", "CT finding (will flag as PUI)", "IRB Required Testing"."

IV. Comments from Reviewer #3:

1. **Concern #1:** The overall presentation and results seems interesting. What is the motivation of the proposed work? Research gaps, objectives of the proposed work should be clearly justified

   Thank you for the suggestion! Our motivation, objective, and research gaps are included in the Abstract and Introduction sections.

   ABSTRACT
   Objective

   > "Despite the high secondary attack rate of COVID-19 in shared households, some exposed individuals do not contract the virus. In addition, little is known about whether the occurrence of COVID-19 resistance differs among people by health characteristics as stored in the electronic health records (EHR). In this retrospective analysis, we develop a statistical model to predict COVID-19 resistance in 8,536 individuals with prior COVID-19 exposure using demographics, diagnostic codes, outpatient medication orders, and count for Elixhauser comorbidities in EHR data from the COVID-19 Precision Medicine Platform Registry."

   INTRODUCTION
   Motivation

   > "Knowing the factors that contribute to an individual's resistance to COVID-19 may facilitate our understanding of the mechanism of viral infection and disease progression. The presence or absence of immune response phenotypes among COVID-19 resistant people may also provide clues to the pathogenesis of COVID-19. In addition, identifying mechanisms of resistance may help the research community to identify potential therapeutic targets to treat COVID-19."

   > "It is also important to distinguish between individuals resistant to infection and those who get infected but remain asymptomatic."

"Therefore, it is important to be able to classify individuals as COVID-19-resistant or non-resistant with high prediction power."

Objective

"To address the gaps mentioned therein, our study aims to (1) explore a broader range of phenotypes that may be associated with COVID-19 resistance on a large scale (2) construct and train machine learning models to predict COVID-19 resistance."

Research Gaps

"Although there have been many studies assessing risk factors for various levels of severity of COVID-19, to our knowledge, there have been few published reports on resistance to SARS-CoV-2 infection. Several independent studies have determined that individuals with blood type O may be less susceptible to SARS- CoV-2 infection. Another group has examined the relationship between HLA haplotypes and susceptibility to COVID-19..."

"However these studies did not assess the exposure to the virus among the tested individuals and performed their analyses comparing the rates of positive test results among different blood types or HLA haplotype cohorts."

"A recent multicenter study assessed the susceptibility to SARS-CoV-2 infection based on polymorphism in the ACE2 receptor, a well-known 'port of entry' for the virus into human cells. The major limitation of this study, however, was the lack of data on clinical outcomes at the population scale."

2. **Concern #2:** Insert a figure demonstrating the overall steps involved.

Thank you for the suggestion! We have added the following workflow graph for the overall process as Figure 1 in the "Materials and Methods" section.

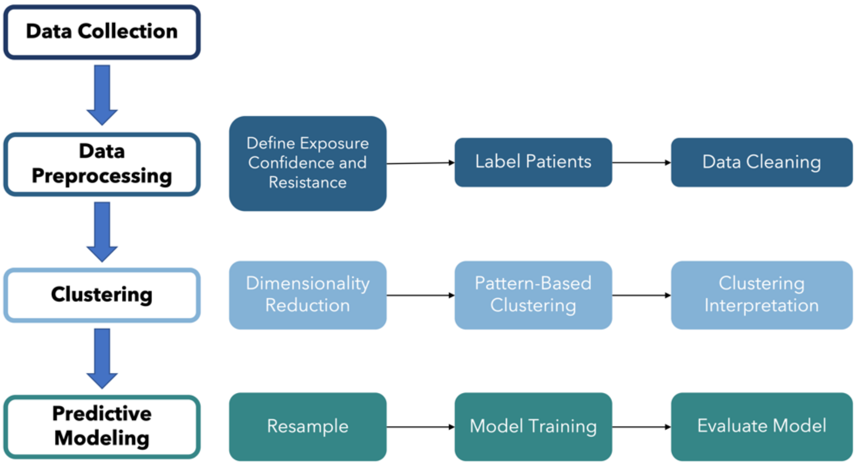

3. **Concern #3:** This is a classification problem, so make a table summarizing the Accuracy, sensitivity, specificity, adn other performance metrics.

Thank you for the suggestion. We have rerun the previously trained model and created a performance comparison table (Table 3) that includes all of the performance metrics that reviewer 3 has raised, including accuracy, sensitivity, and specificity.

	XGBoost		Logistic Regression		Random Forest	
	Testing	HHI Testing	Testing	HHI Testing	Testing	HHI Testing
**AUROC**	0.61	0.62	0.57	0.50	0.60	0.53
**Accuracy**	0.63	0.77	0.77	0.77	0.49	0.78
**Sensitivity**	0.64	0.79	0.80	0.85	0.48	0.85
**Specificity**	0.57	0.63	0.32	0.25	0.69	0.38

4. **Concern #4:** Authors used XGBoost model for model development. Authors are requested to compare XGboost algorithm performance with traditional algorithms and make a comparative study. Authors are suggested to include more discussion on the results and also include some explanation regarding the justification to support why the proposed method is better in comparison towards other methods

We considered a variety of models to use as our primary model, and we chose XGBoost because of its superior performance against the other models in our tests. After hyperparameter tuning all of these models as well as we could through five-fold cross-validation, the XGBoost model performed the best on our held-out low-confidence exposure cohort (testing set) and the HHI testing subsets. We realize we mentioned this in our results and not in our methods, but the XGBoost model performed the best on our held-out low-confidence exposure cohort and the HHI testing subsets, which is why it was selected for our SHAP analysis. We added that to our methods to make this more explicit. We also added more details (as shown below) to our Results section to explain how we considered all of the performance metrics together to arrive at our decision to choose XGBoost due to its balanced and consistently high performance. Thank you for catching that.

> "The logistic regression model had higher accuracy and sensitivity than XGBoost, but it's much lower AUROC and specificity indicated that it may be biased in favor of making positive predictions. The XGBoost model performed consistently well across all metrics, leading us to select it for further analysis."

We believe we could further justify this choice by including more information about how XGBoost goes beyond logistic regression and random models forest models by correcting itself over time by increasing the weight of high-error points to improve its fit. This can sometimes lead to overfitting, but by testing it on two held-out testing sets, one of which was sampled from a population different from the one we trained the models on, we verified that it likely wasn't overfitting. We have added the above to our Discussion section of our revision as shown below and thank you for your advice:

> "We believe the XGBoost performed the best because it goes beyond logistic regression and random models forest models by correcting itself over time by increasing the weight of high-error points to improve its fit. This can sometimes lead to overfitting, but by testing it on two held-out testing sets, one of which was sampled from a population different from the one we trained the models on, we verify that it likely wasn't overfit. However, it still wasn't a very reliable predictor despite all these efforts."

5. **Concern #5:** Whether hyperparameter tuning performed? If yes what strategy is followed to select the best hyperparameters?

Hyperparameter tuning using grid search over various hyperparameters was performed to select the best hyperparameters for each of the machine learning models we have trained. We tried a variety of parameters for each model to help tune the bias-variance tradeoff by determining the optimal optimization and regularization techniques that fit the data closely enough to be a good fit while not overfitting them enough to reduce the cross-validation validation AUROC. We didn't mention the specific hyperparameters we tried, but we agree that it is important to include, so we thank you for your advice and have added the following description to our Results section:

> "The five-fold cross validation hyperparameter tuning yielded the following models. For XGBoost, learning rates from 0.01 to 0.5, between 5 and 100 estimators, and the boosters gbtree, gblinear, and dart were performed. The learning rate of 0.5, 10 estimators, and the booster gbtree were selected for producing the highest cross-validation AUROC. For logistic regression, the solvers Liblinear, Newton Conjugate Gradient, and LBFGS were tried. The regularizers L1, L2, and elastic net were tried, and inverse regularization strengths from e^-5 to e^2 were tried. The Liblinear solver, L2 penalty, and e^1.59 inverse regularization strength were selected for producing the highest cross-validation AUROC. For random forest (RF), we examined max depths from 10 to 100, min leaf samples from 1 to 4, min samples for a split from 2 to 10, and class weights that were balanced, balanced subsample, or none. A max depth of 15, 1 min leaf sample, 4 samples in a split, and no class weights were selected for producing the highest cross-validation AUROC."

6. **Concern #6:** Results and discussion section should be improved.

We used all the advice you gave in Concern #3,#4,#5,#7 to add to and improve on the Results and Discussion sections. We hope these additions satisfy your concerns regarding our inadequate results and discussion sections.

7. **Concern #7:** Discuss about the correlation between the variables/features considered.

Thank you for bringing this up, we realize that this is an important concept to discuss in informatics papers and should have included this. While we don't directly remove correlated features, our recursive feature elimination removes features that provided redundant information to the model, which includes some correlated features. However, there are instances when including correlated features is important. For example, it's possible for two medications to perform similar functions and be conditionally correlated given some baseline conditions, but both medications can still be impactful to the model because patients generally only take one or the other and we don't want to remove an important population that take the one we remove. We use the broadest medication and diagnostic classes to group similar codes to avoid this type of correlation, but across coding groups, correlated features are removed or included according to whether or not they benefit the model through recursive feature elimination. We have added this to our Discussion section as shown below:

> "We examined feature correlation to avoid missing important features due to variables contributing the same information, but we didn't want to remove features that improved model performance. Our recursive feature elimination removes features that provided redundant information to the model, which includes some correlated features. We also use the broadest medication and diagnostic classes to group similar codes."

8. **Concern #8:** Explain why the current method was selected for the study, its importance and compare with traditional methods.

We agree that it is important to properly justify the model we used, and we hope we sufficiently addressed this in Concern #4 you have raised.

9. **Concern #9:** Does this kind of study have never attempted before? Justify this statement and give an appropriate explanation to do so in this paper.

To the author's knowledge, this kind of study has never been attempted before. We have included that in the Introduction section of the original manuscript.

> "To the authors' knowledge, this will be the first study to consider exposure while defining the cases of resistance and predict COVID-19 resistance using machine learning approaches."

Below are some statements we had in our original manuscript that have explained and justified why we believe this kind of study has never been attempted before:

- "Although there have been many studies assessing risk factors for various levels of severity of COVID-19, to our knowledge, there have been few published reports on resistance to SARS-CoV-2 infection. Several independent studies have determined that individuals with blood type O may be less susceptible to SARS- CoV-2 infection. Another group has examined the relationship between HLA haplotypes and susceptibility to COVID-19..."
- "However, these studies did not assess the exposure to the virus among the tested individuals and performed their analyses comparing the rates of positive test results among different blood types or HLA haplotype cohorts."
- "A recent multicenter study assessed the susceptibility to SARS-CoV-2 infection based on polymorphism in the ACE2 receptor, a well-known 'port of entry' for the virus into human cells. The major limitation of this study, however, was the lack of data on clinical outcomes at the population scale."
- "To address the gaps mentioned therein, our study aims to (1) explore a broader range of phenotypes that may be associated with COVID-19 resistance on a large scale (2) construct and train machine learning models to predict COVID-19 resistance."

10. **Concern #10:** Quality of figures is so important too. Please provide some high-resolution figures. Some figures have a poor resolution.

We apologize for the poor resolution of some of our initial figures submitted. Our resubmission includes high-resolution versions of these figures. We thank you for calling this to our attention.
